# Supplementary material for: Elucidating the Role of Santalol as a Potent Inhibitor of Tyrosinase: In Vitro and In Silico Approaches
Source: Molecules. 2022 Dec 15;27(24):8915. doi: 10.3390/molecules27248915 (PMC9786741; doi:10.3390/molecules27248915)
Supplement: Supplementary file 1 [file molecules-27-08915-s001.zip › molecules-2042120-supplementary.pptx]

## Slide 1
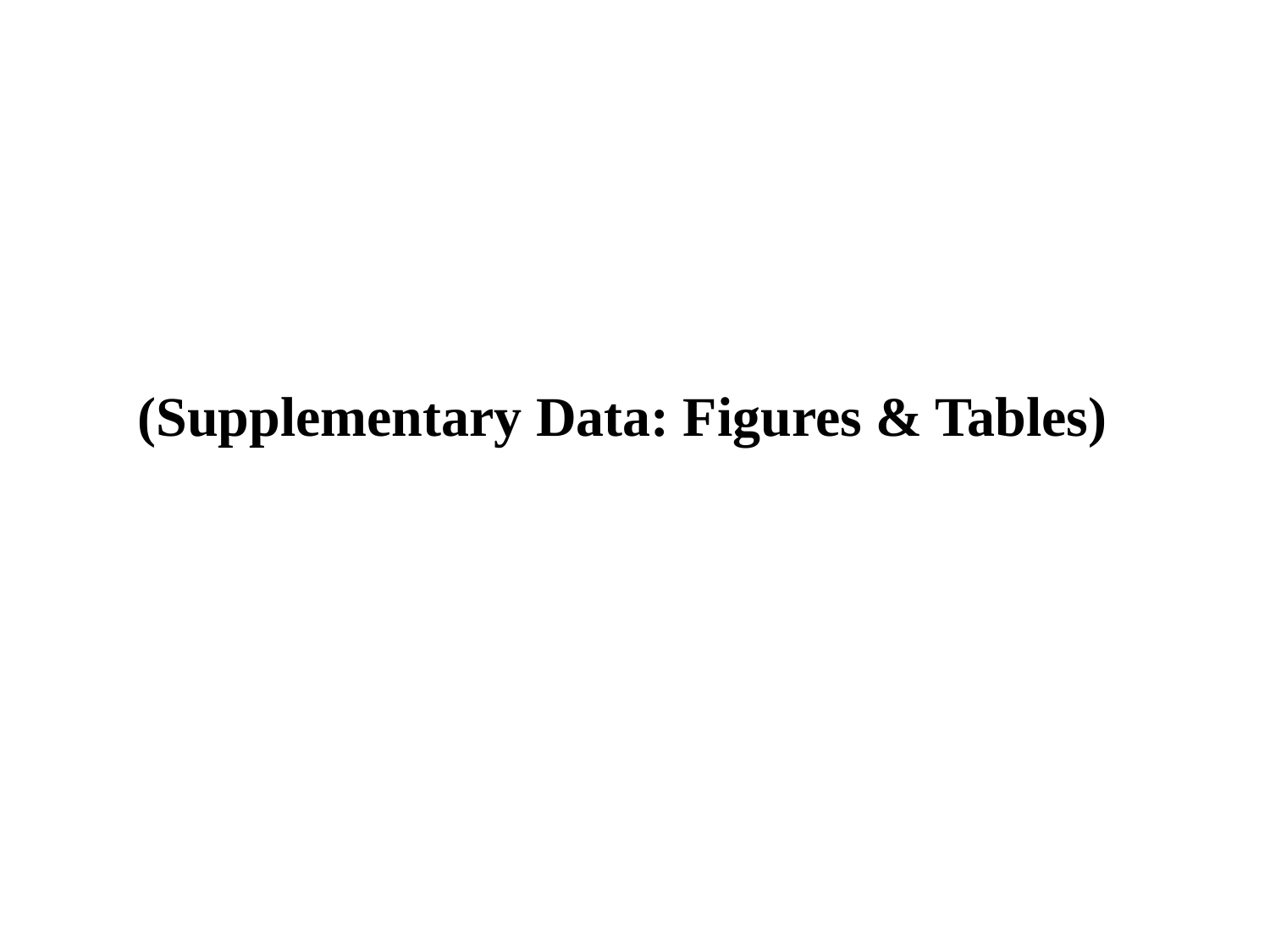

(Supplementary Data: Figures & Tables)

## Slide 2
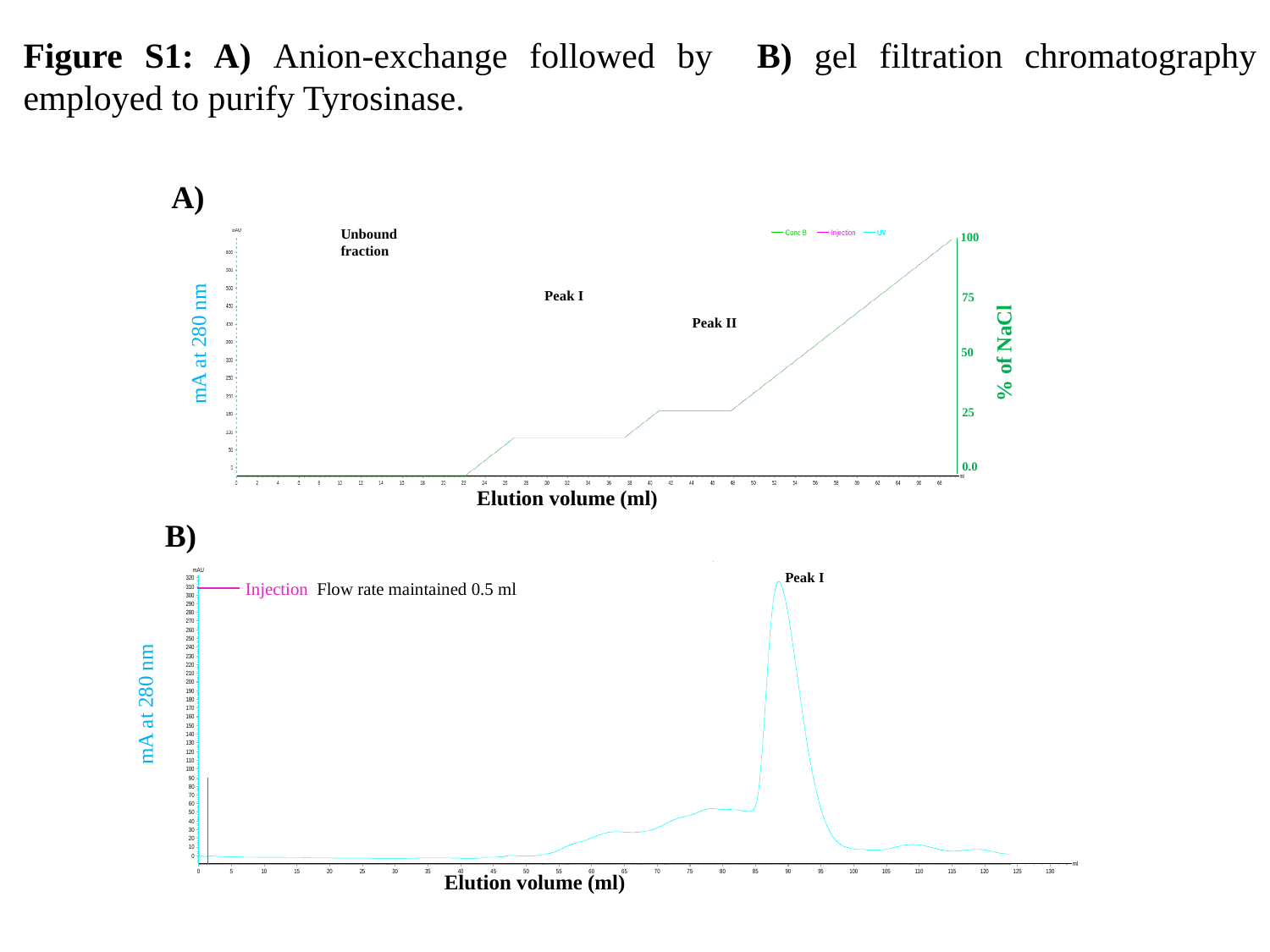

Figure S1: A) Anion-exchange followed by B) gel filtration chromatography employed to purify Tyrosinase.
A)
mA at 280 nm
Unbound fraction
100
Peak I
75
% of NaCl
Peak II
50
25
0.0
 Elution volume (ml)
B)
mA at 280 nm
Peak I
Injection Flow rate maintained 0.5 ml
 Elution volume (ml)

## Slide 3
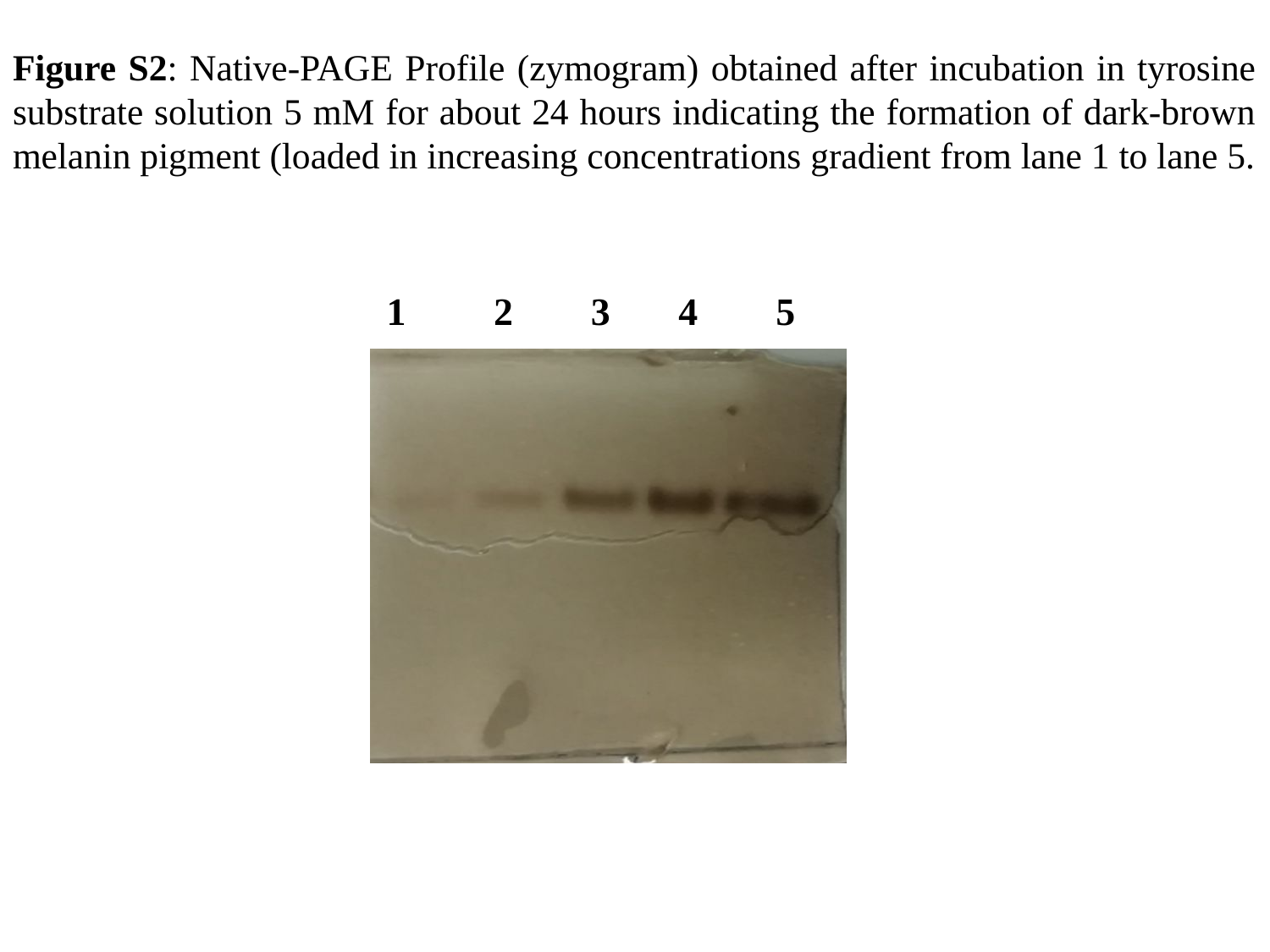

# Figure S2: Native-PAGE Profile (zymogram) obtained after incubation in tyrosine substrate solution 5 mM for about 24 hours indicating the formation of dark-brown melanin pigment (loaded in increasing concentrations gradient from lane 1 to lane 5.
1 2 3 4 5

## Slide 4
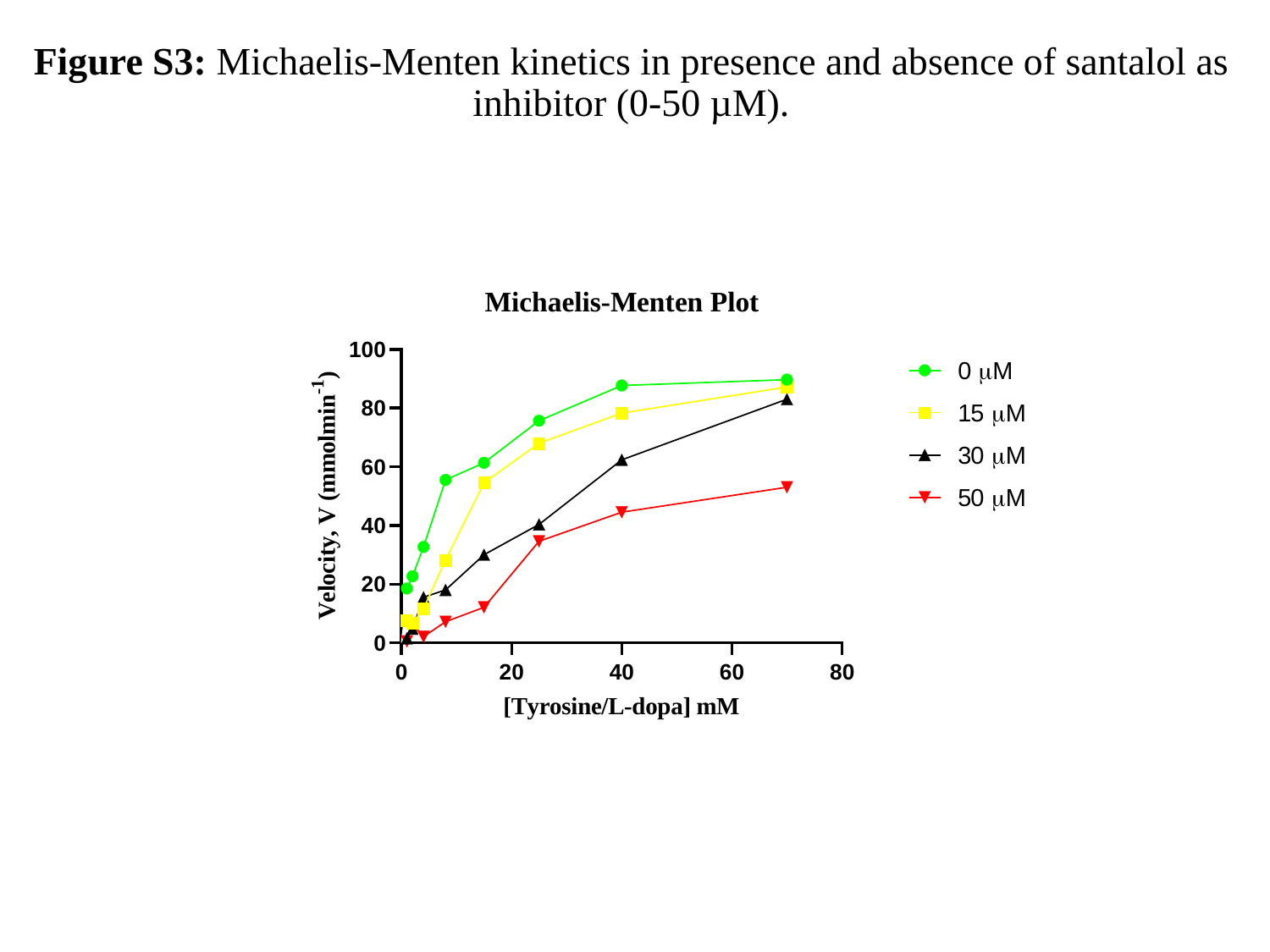

# Figure S3: Michaelis-Menten kinetics in presence and absence of santalol as inhibitor (0-50 µM).

## Slide 5
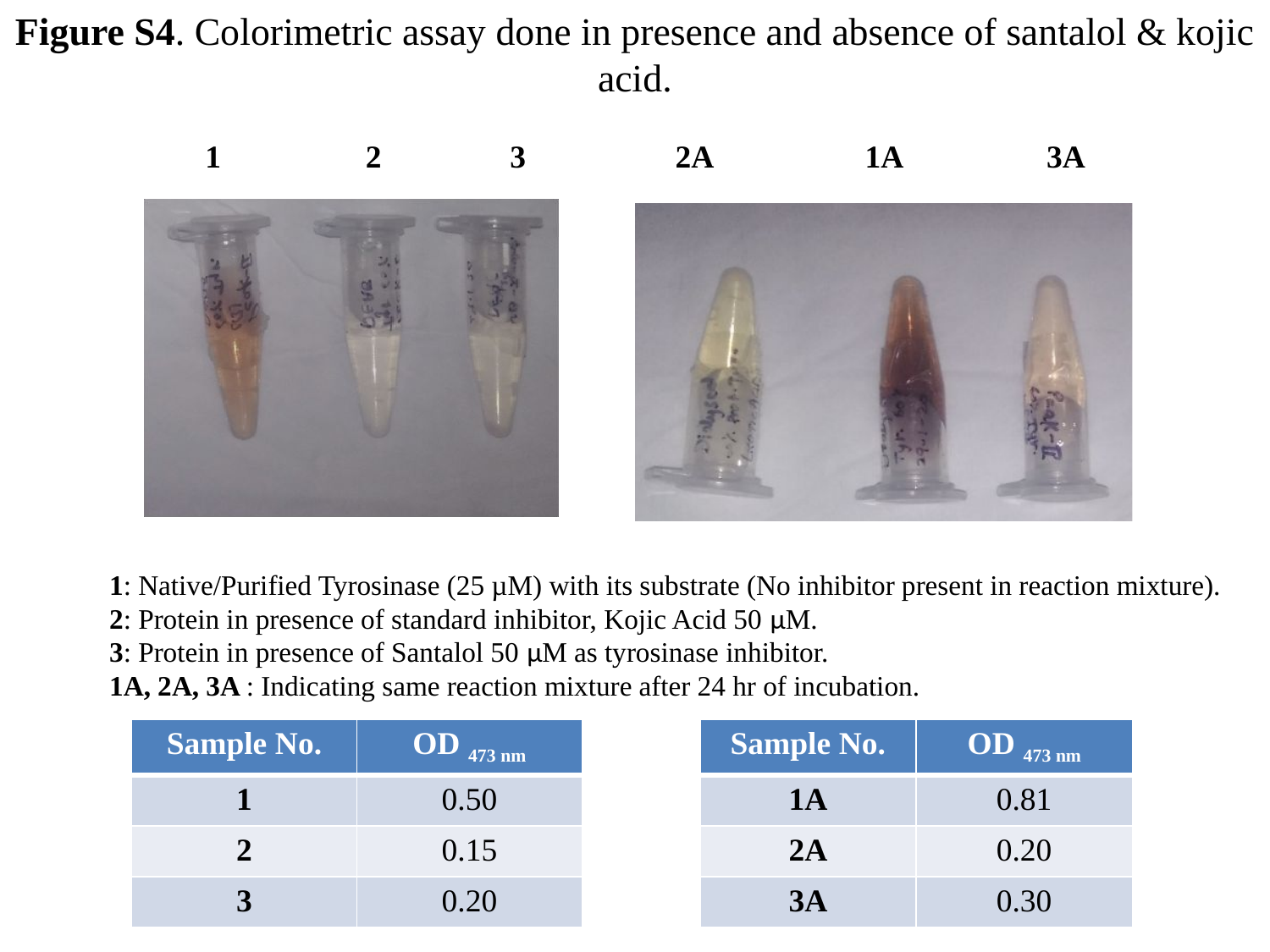

Figure S4. Colorimetric assay done in presence and absence of santalol & kojic acid.
 1 2 3
 2A 1A 3A
1: Native/Purified Tyrosinase (25 µM) with its substrate (No inhibitor present in reaction mixture).
2: Protein in presence of standard inhibitor, Kojic Acid 50 µM.
3: Protein in presence of Santalol 50 µM as tyrosinase inhibitor.
1A, 2A, 3A : Indicating same reaction mixture after 24 hr of incubation.
| Sample No. | OD 473 nm |
| --- | --- |
| 1 | 0.50 |
| 2 | 0.15 |
| 3 | 0.20 |
| Sample No. | OD 473 nm |
| --- | --- |
| 1A | 0.81 |
| 2A | 0.20 |
| 3A | 0.30 |

## Slide 6
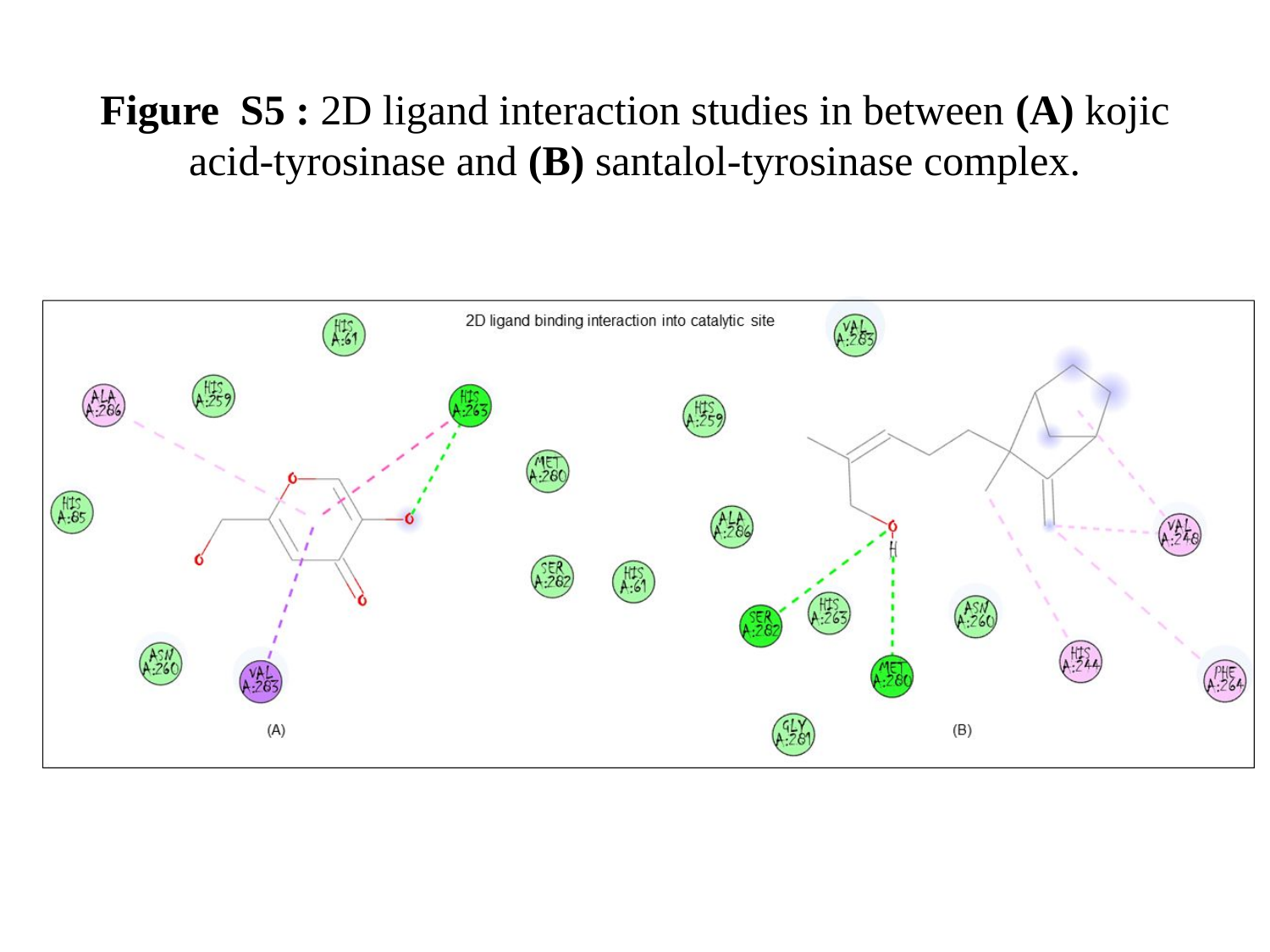

# Figure S5 : 2D ligand interaction studies in between (A) kojic acid-tyrosinase and (B) santalol-tyrosinase complex.

## Slide 7
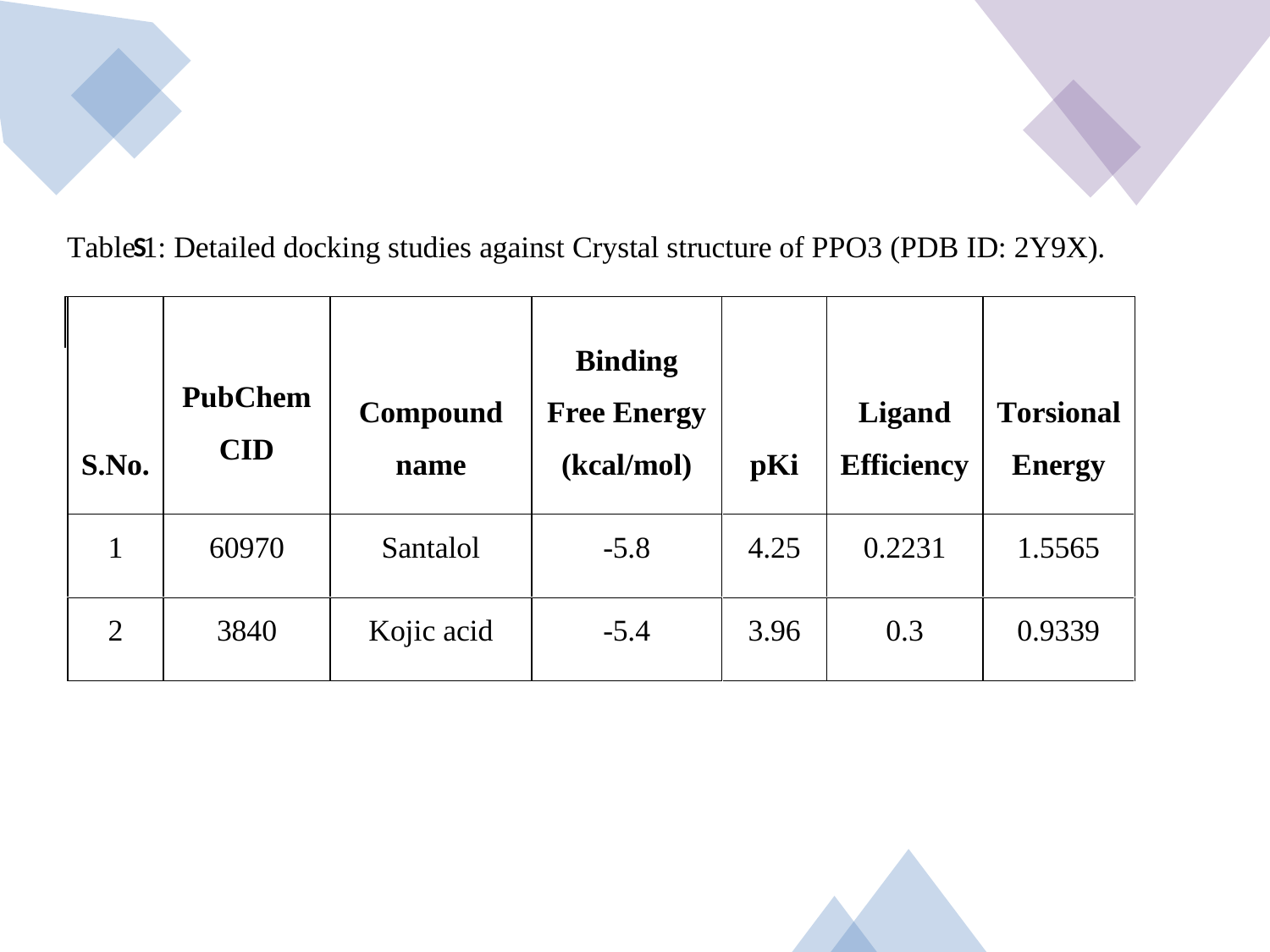

S
| |
| --- |

## Slide 8
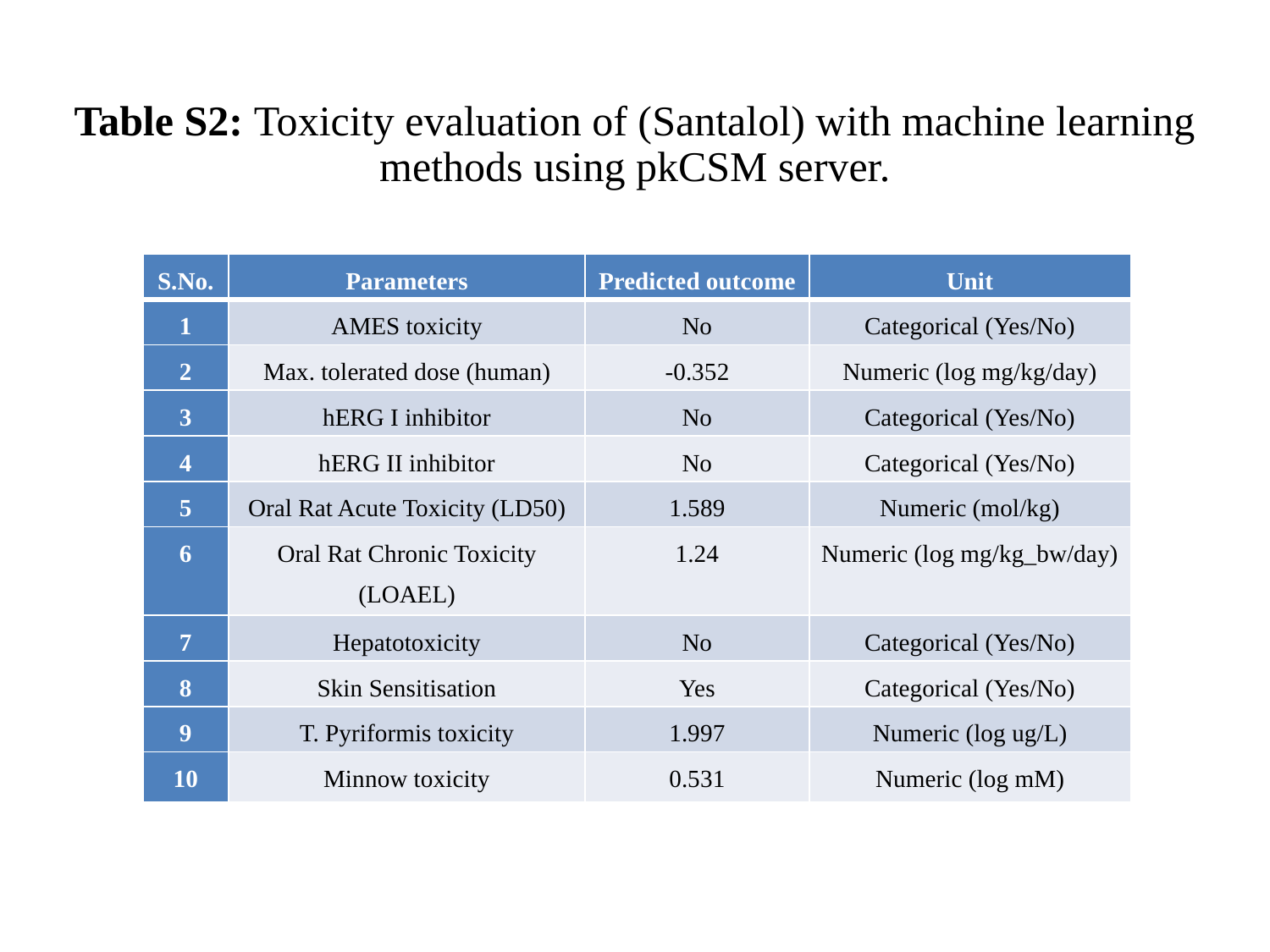

Table S2: Toxicity evaluation of (Santalol) with machine learning methods using pkCSM server.
| S.No. | Parameters | Predicted outcome | Unit |
| --- | --- | --- | --- |
| 1 | AMES toxicity | No | Categorical (Yes/No) |
| 2 | Max. tolerated dose (human) | -0.352 | Numeric (log mg/kg/day) |
| 3 | hERG I inhibitor | No | Categorical (Yes/No) |
| 4 | hERG II inhibitor | No | Categorical (Yes/No) |
| 5 | Oral Rat Acute Toxicity (LD50) | 1.589 | Numeric (mol/kg) |
| 6 | Oral Rat Chronic Toxicity (LOAEL) | 1.24 | Numeric (log mg/kg\_bw/day) |
| 7 | Hepatotoxicity | No | Categorical (Yes/No) |
| 8 | Skin Sensitisation | Yes | Categorical (Yes/No) |
| 9 | T. Pyriformis toxicity | 1.997 | Numeric (log ug/L) |
| 10 | Minnow toxicity | 0.531 | Numeric (log mM) |
